# H19 regulates trophoblastic spheroid adhesion by competitively binding to let-7

**DOI:** 10.1530/REP-18-0339

**Published:** 2019-02-18

**Authors:** Dongmei He, Hong Zeng, Jingfei Chen, Lan Xiao, Yuhao Zhao, Nenghui Liu

**Affiliations:** 1 Reproductive Medicine Center, Xiangya Hospital, Central South University, Changsha, China; 2 Department of Obstetrics and Gynecology, Xiangya Hospital, Central South University, Changsha, China

## Abstract

*Integrin β3* (*ITGB3*), which is the target gene of the miRNA let-7 that can be antagonized by long noncoding RNA (lncRNA) H19, is well known to have a critical role in endometrium receptivity. However, the regulation of *ITGB3* in cell–cell or cell–extracellular matrix adhesion and invasion for the maintenance of early pregnancy remains unknown. This study aimed to explore the role of the H19/let-7/ITGB3 axis in regulating trophoblastic spheroid adhesion and *in vitro* invasion ability using the HTR-8/SVneo cell line and to investigate the expression levels of lncRNA H19 and *ITGB3* in human products of conception. The *in vitro* knockdown of H19 resulted in decreased expression of *ITGB3* at the mRNA and protein levels and reduced the adhesion and invasion ability. In the embryonic chorion tissue of spontaneous abortion (SA), the expressions of H19 and *ITGB3* at both the mRNA and protein levels decreased. The results of quantitative RT-PCR, Western blot analysis, dual-luciferase report gene and functional miRNA let-7 rescue experiments, adhesion assay and *in vitro* transwell invasion assay confirmed that H19 regulated trophoblastic spheroid adhesion with endometrial stromal cells through the H19/let-7/ITGB3 axis, thereby providing an improved understanding of the molecular mechanism of SA.

## Introduction

In all clinically confirmed pregnancies, the incidence of spontaneous abortion (SA) is approximately 15%. More than 80% of abortions occur within 12 weeks of pregnancy; thereafter, the rate of abortion rapidly declines. Successful human embryo implantation, which requires a viable blastocyst and uterine receptivity, involves a complex and critical multistep process, including embryo apposition/adhesion, followed by penetration and invasion. The initial but fundamental steps of embryo implantation are feto–maternal interaction and cell adhesion and invasion between the blastocyst and endometrial luminal epithelial cells.

Although the regulatory mediators have already been identified for mouse embryo implantation, the mechanism of human embryo implantation remains unclear. Ethical concerns and technical constraints have led to many obstacles in human subjects (Hannan *et al.* 2010). An *in vitro* adhesion assay using human endometrial stromal cells (HESCs) and simulated trophoblast spheroid was performed to clarify the adhesion process during embryo implantation. This study investigated the effects of both the downregulation and overexpression of H19 on the adhesion and invasion ability of the HTR-8/SVneo cell line, which was derived from human first-trimester extravillous trophoblast (EVT). The lncRNA H19, which is mainly located in the cytoplasm and measures approximately 2.3-kb long, is maternally expressed and had been hypothesized by many to be involved in posttranscriptional regulation (Milligan *et al.* 2002). A large number of investigations focused on the role of H19 in the initiation and development of carcinoma (Matouk *et al.* 2013). One study suggested the important role of H19 in trophoblast physiology, based on the findings of high H19 expression in human placental intermediate trophoblasts and cytotrophoblasts (Ariel *et al.* 1994). Polymorphisms in IGF2/H19 gene locus are associated with platinum-based chemotherapeutic response in Chinese patients with epithelial ovarian cancer (Zeng *et al.* 2019). Long non-coding RNA H19 promotes TDRG1 expression and cisplatin resistance by sequestering miRNA-106b-5p in seminoma (Wei *et al.* 2018). Regulation of tumor cell migration and invasion by the H19/let-7 axis is antagonized by metformin-induced DNA methylation (Yan *et al.* 2015). Aberrant methylation of the H19-imprinting control region may increase the risk of SA (Liu *et al.* 2018). H19 lncRNA can act as a sponge that binds and regulates the functions of specific miRNAs. H19 expression had been reported to increase the level of the miRNA-processing enzyme Dicer, which targets miRNA let-7 (Kallen *et al.* 2013). H19 at positions 1244 and 1617 contains functional let-7-binding sites and functions as a molecular sponge for the major let-7 family of miRNAs (Kallen *et al.* 2013). By binding to imperfect complementary sequences in mRNAs, miRNA Let-7 regulates target gene expression by translational repression or mRNA destabilization. In mice, miRNA let-7a was found to inhibit embryo implantation by decreasing the expression of the target gene integrin beta 3 (*ITGB3*) (Liu *et al.* 2012). However, evidence on human embryo implantation of H19 had been scarce.

Integrins are located on the cell surface and have a fundamental role in cell–cell and cell–extracellular matrix adhesion. A previous study showed that alterations in *ITGB3* expression were strongly associated with the acquisition of invasive and/or metastatic properties (Li *et al.* 1998), but only little is known about the biochemical mechanisms that regulate the *ITGB3* gene expression in cells. In postimplantation processing, *ITGB1* had been thought to be the critical gene in embryo implantation and placental development (Cross *et al.* 1994). Aside from producing proteinase to degrade extracellular matrix, trophoblasts also change the expression of adhesion molecules during the implantation process (Cross *et al.* 1994). The type of integrins expressed by cytotrophoblasts in the process of embryo implantation gradually changes. Some integrins can promote and some may inhibit the implantation of cytotrophoblasts (Cross *et al.* 1994). Embryonic spheroid attachment had been shown to be reduced by the downregulated expression of the ITGB3 protein (Zhu *et al.* 2013). Most studies explored integrin 3 on endometrial cells as a sign of opening of the endometrium receptive window (Haouzi *et al.* 2009). The downregulation of *ITGB3* impairs endometrial receptivity and leads to SA (Germeyer *et al.* 2014) and infertility (Ceydeli *et al.* 2006). SA is a common complication of pregnancy. The possibility for a woman to experience at least one sporadic pregnancy loss in her life is 25–50%, and more than 80% of SAs take place in the first trimester (i.e., within 12 weeks of gestation) (Rai & Regan 2006, Saravelos *et al.* 2010). A previous study showed that the target gene of let-7, *ITGB3*, was a biological marker of good endometrial receptivity and was crucial in blastocyst implantation. Another study showed that the expressions of H19 lncRNA and ITGB3 protein were downregulated in the endometrium of patients with recurrent implantation failure (Zeng *et al.* 2017). Based on the above theories, we aimed to explore the role of the H19/let-7/ITGB3 axis disorder in early pregnancy loss.

The present study investigated the effects of both *in vitro* downregulation and overexpression of H19 on trophoblast adhesion and invasion ability. We explored the expression levels of H19 and *ITGB3* in human chorionic villous tissues from normal pregnancies and SAs between 6 and 10 weeks of gestation. Furthermore, we postulated that the H19/let-7/ITGB3 axis correlated with the molecular mechanisms of SA. The findings might be valuable for future studies on SAs.

## Materials and methods

### Cell culture

HTR-8/SVneo, which we called HTR-8 in this study and is a cell line derived from human first-trimester EVT, was bought from the American Type Culture Collection with STR identification report and cultured in Roswell Park Memorial Institute 1640 medium (BI, kibbutz Beit Haemek, Israel) supplemented with 10% fetal bovine serum (FBS) (BI, kibbutz Beit Haemek, Israel). Human endometrium stromal cells (HESCs), an endometrium cell line derived from the stromal cells, were obtained from an adult female with myomas, which were generously donated by Lan Xiao (Central South University, Changsha, China) and cultured in a 1:1 mixture of phenol red-free Dulbecco’s minimum essential medium/F12 (Gibco) supplemented with 10% FBS. Both cell lines were cultured with 100 IU/mL penicillin and 100 µg/mL streptomycin (BI), then incubated in 5% carbon dioxide at 37°C.

### Cell transfection

A lentiviral vector with H19 knockdown and overexpression and a lentiviral vector alone, which was used as a negative control, were constructed by Genechem (Shanghai, China). Cells with H19 knockdown were defined as shH19 group and the corresponding control group was called shCon. The nucleotide sequences of the three pairs of double-stranded shRNA fragments were based on the sequences of the *H19* gene to construct an interfere vector and included the following: shH19-1: 5′-CCGGCAGCCTTCAAGCATTCCATTACTCGAGTTTTTG-3′; shH19-2: 5′-CCGGCAGGAGAGTTAGCAAAGGTGACTCGAGTTTTTG-3′; and shH19-3: 5′-CCGGAACCCACAACATGAAAGAAATCTCGAGTTTTTG-3′, the scramble sequence of shCon was 5′-CCGGTTCTCCGAACGTGTCACGTTTTTTG-3′. Cells that overexpressed H19 were defined as the PH19 group and the corresponding control group was called Pvector. Cells of the HTR-8 were seeded in six-well plates at 40% confluence on the day before transfection. HTR-8 cells were transfected at the proper multiplicity of infection. Green fluorescent protein expression was used to assess the infection efficiency after 96 h of infection. RT-qPCR was performed to evaluate the efficiency of H19 expression.

### Spheroid formation and adhesion assay

To perform trophoblastic spheroid formation assay, approximately 1 × 10^3^ HTR-8 cells/well were seeded into ultra-low-attachment, 96-well flat bottom plates (Corning, NY, USA), using the same cell culture media as described earlier for 24 h. The trophoblastic spheroids were used to mimic a human blastocyst. Approximately 40 trophoblast spheroids were seeded to a six-well plate covered with HESCs in advance, and the number of trophoblastic spheroids was counted under a microscope. The bottom of the six-well plate was marked into six fan-shaped areas in advance, in order to make counting easy. The cells were rinsed twice with 1× phosphate-buffered saline (BI), in order to remove debris and the unattached spheroids after 24 h of co-culture. The remaining attached spheroids in the six-well plate were counted. The attachment ratio was calculated as follows: number of remaining attached spheroids/number of seeded spheroids.

### Transwell invasion assay

HTR-8 cells that were transfected with sh19 and PH19 were trypsinized with the respective control cells, and the supernatant was removed by centrifugation. The cells were resuspended in a serum-free medium, and the number of cells required was counted according to the cell count and plate count. A 100-μL cell suspension of 1 × 10^5^ cells was added above the chamber and 500 μL of culture medium containing 10% FBS was added to the lower chamber. After ensuring that no air bubbles were generated between the lower chamber culture medium and the chamber, the mixture was cultured in the incubator for 24 h. The cells were fixed in 4% paraformaldehyde for 30 min. Thereafter, the cells and Matrigel were removed from the upper surface of the membrane with a cotton bud and stained with 0.1% crystal violet for 30 min. The number of cells on the underside was determined using light microscopy. Five randomly selected fields were counted per insert.

### Patients

This study was conducted between October 10, 2017 and February 20, 2018 at the obstetrical clinic of the First Affiliated Hospital of Central South University in Hunan, China. Fresh tissue samples were collected and processed within 10 min. Each sample was divided into two parts and was snap-frozen in liquid nitrogen before storage −80°C. The inclusion criteria for selecting patients and collecting embryonic chorionic tissue samples were as follows: (1) regular menstrual cycle of 28–35 days; (2) ≤35 years of maternal age; (3) no previous history of SA and (4) 6 weeks of gestation. Patients who were overweight or obese and those with polycystic ovarian syndrome; endometriosis; uterine malformations, such as bicornuate uterus and uterine cavity adhesion; thyroid dysfunction; ovarian tumor; and embryo chromosomal abnormalities were excluded from this study. Patients who experienced their first SA were assigned as the case group (*n* = 12). Mothers with no previous history of SA and asked for an elective termination of pregnancy were assigned as the control group (*n* = 12). The data did not contain any information that could identify the patients. All patients provided written informed and ethical consent, and the project was approved by the Ethics Committee of Xiangya Hospital.

### RT-qPCR

The total RNA isolated from 50 mg of embryonic chorionic tissues or cultured from the HTR-8/SVneo cell line was extracted using a standard TRIzol-based protocol (Invitrogen), according to the manufacturer’s instructions. The total RNA (1 µg) of each sample was reverse-transcribed using a TransScript One-Step gDNA Removal and cDNA Synthesis SuperMix Kit (TransGen Biotech, Beijing, China), which could eliminate genomic DNA contamination. cDNA was synthesized in a 20-µL reaction mixture containing 1 µg of total RNA. Real-time qPCR was performed in a 20-µL reaction mixture using a TransStart Tip Green qPCR SuperMix Kit (TransGen Biotech). The melting curve was used to verify the specificity. The relative RNA expression level was analyzed using the method of 2^(−ΔΔCt)^. The oligonucleotide sequences designed for amplifying genes and the size of the amplified fragments are described in Table 1.

**Table 1:** The primer sequences of gene involved in the study are displayed as follows.

Gene	Sequence (5′–3′)		Amplicon length (bp)
	Forward	Reverse	
*H19*	CGTGACAAGCAGGACATGACA	CCATAGTGTGCCGACTCCG	146
*ITGB3*	GAGCCCTACATGACGAAAATACC	CTTGCCAGTGTCCTTAAGCTCT	83
*ACTB*	GCACCACACCTTCTACAATGAG	GATAGCACAGCCTGGATAGCA	165

### Reagents and antibodies

Rabbit monoclonal antibodies against ITGB3 (#4702) was purchased from Cell Signaling Technology. Mouse monoclonal antibodies against β-actin (#AC010), glyceraldehyde-3-phosphate dehydrogenase (GAPDH) (#AC002) and horseradish peroxidase-conjugated secondary antibodies were purchased from ABclonal Technology, China.

### Western blot analysis

The total protein isolated from 50 mg or embryonic chorionic tissues or cultured from the HTR-8/SVneo cell line was extracted using the Radio-Immunoprecipitation Assay (Servicebio, Wuhan, China) lysis solution, according to the manufacturer’s instructions. After centrifugation, the supernatant was collected, and the protein concentrations were determined using the Bradford assay (DingGuo, Beijing, China). Thereafter, the total protein of each sample was mixed with a 5× SDS loading buffer in a 4:1 proportion and was boiled at 95°C for 10 min. For the Western blot assay, 30 μg of each protein sample was separated by 10% SDS–polyacrylamide gel electrophoresis, followed by electrotransfer onto a 0.45 µm PVDF (Millipore). The membrane was blocked by incubation with 5% non-fat dried milk in 0.1% Tris-buffered saline with Tween 20 (TBST) for 1 hour. Subsequently, the membrane was incubated overnight with monoclonal primary antibodies specific to ITGB3 (dilution: 1/1000), GAPDH (dilution: 1/5000) and β-tubulin (dilution: 1/5000). The following day, the membrane was washed with TBST for three times for 5 min each time. Subsequently, the membrane was incubated with the respective secondary antibodies for 1 hour before washing, as described earlier. The protein bands were detected using hypersensitive enhanced chemiluminesence (servicebio, Wuhan, China) in a dark chamber. The integrated light density and gray values were calculated using ImageJ software (National Institutes of Health, Bethesda, MD, USA) and were normalized against the bands obtained for GAPDH or β-actin.

### Luciferase reporter system detection of the target gene

A double luciferase reporter gene was used to investigate whether human miRNA let-7a-5p can regulate the expression of the human *ITGB3* gene. The PCR primers were designed based on the human *ITGB3* gene 3′-UTR sequence information. The 3′-UTR sequences of the human *ITGB3* gene were amplified by PCR using the genomic DNA of the 293T cell as a template and were cloned into pmiR-RB-REPORTTM dual luciferase. The Renilla luciferase gene was used as the reporter fluorescence, whereas the Firefly luciferase gene was used as the internal reference gene in the reporter vector. The regulatory effect of human miRNA let-7a-5p on the human *ITGB3* gene was determined based on the relative changes in Renilla luciferase activity.

### Statistical analysis

Values were reported as median (interquartile range) for non-normally distributed variables, mean ± s.d. for normally distributed variables or percentage. The differences in continuous variables between the intervention and control groups were analyzed using the independent samples *t*-test. Spearman correlations were performed for gene co-expression analyses. GraphPad prism 7 (GraphPad Software) was used for data analysis. A *P* value <0.05 indicated statistical significance.

## Results

### H19 regulated the expression of *ITGB3* by competitively binding to miRNA let-7

**Figure 1:**
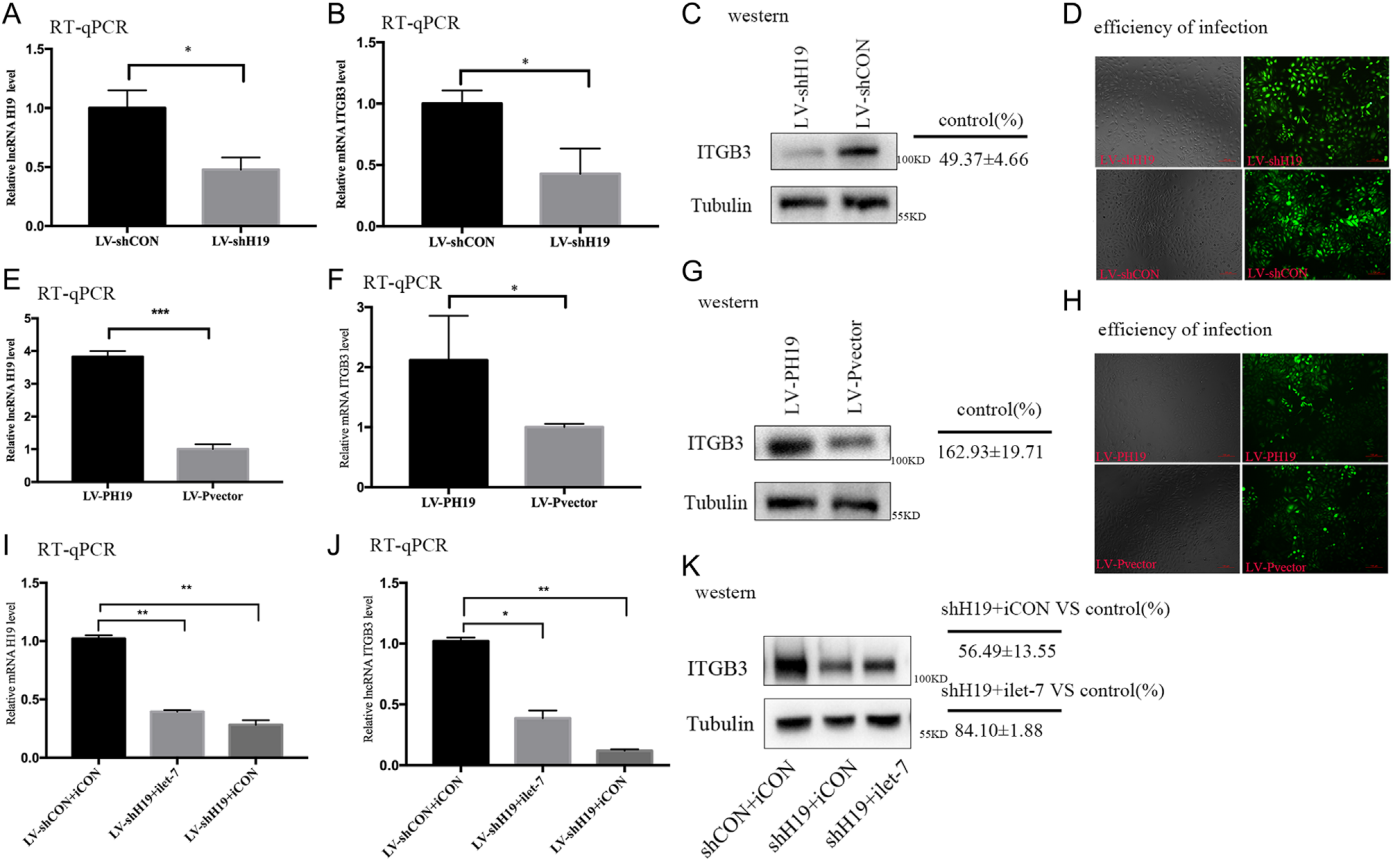
The H19/let-7 axis regulates the expression of integrin β3 (*ITGB3*) in HTR-8. (A, B and C) The HTR-8 cells are transfected with shH19 or shCon. (A) The relative H19 levels after normalization with beta-actin mRNA. (B) The relative *ITGB3* mRNA level is determined using quantitative RT-PCR. (C) The *ITGB3* protein level is analyzed using the Western blot analysis. Protein markers are indicated on the right side of the blots. Group *t*-tests are performed to compare each data point with the control (shCon). Numbers are presented mean ± s.d. (*n* = 3). **P < 0.01. (D,H) The efficacy of infection of these lentiviral vectors under a fluorescence microscope. (E, F and G) The HTR-8 cells are transfected with pH19 or pvector. (E) The relative H19 levels after normalization with beta-actin mRNA. (F) The relative *ITGB3* mRNA level is determined using quantitative RT-PCR. (G) The *ITGB3* protein level is analyzed using the Western blot analysis. Protein markers are indicated on the right side of the blots. Group *t*-tests are performed to compare each data point with the control (pvector). Numbers are presented as mean ± s.d. (*n* = 3). **P < 0.01. (I, J and K) The HTR-8 cells are co-transfected with shCon and iCon (miRNA inhibitor control), shH19 and iLet-7 (let-7-specific inhibitor), or shH19 and iCon. RNAs are extracted 48 h after transfection and analyzed using RT-qPCR. (I) The relative H19 levels after normalization with beta-actin mRNA. (J) The relative *ITGB3* mRNA level is determined using quantitative RT-PCR. (K) Total protein is extracted 48 h after transfection and analyzed using the Western blot analysis. Protein markers are indicated on the right side of the blots. Group *t*-tests are performed to compare each data point with the control (shCon plus iCon). Numbers are presented mean ± s.d. (*n* = 3). **P < 0.01.

HTR-8 cells were transfected with two lentiviral constructs harboring shH19 and PH19 to investigate whether H19 regulated the expression of *ITGB3* by competitively binding to miRNA let-7. In parallel, a negative control was run. The efficacy of infection of these lentiviral vectors was more than 80% under a fluorescence microscope (Fig. 1D and H). Downregulation of H19 decreased the expression of *ITGB3* at both the mRNA and protein levels (Fig. 1A, B and C), whereas H19 overexpression upregulated *ITGB3* at both the mRNA and protein levels (Fig. 1E, F and G). Next, miRNA rescue experiments were performed by co-transfection of let-7 inhibitor (ilet-7) and shH19. Combination of ilet-7 and shH19 (Fig. 1I) partially restored the expression of *ITGB3* both at the mRNA and protein levels (Fig. 1J and K), and the rescue effect appeared to be significant, when compared with that of combination transfection of the let-7 inhibitor control and shH19. Collectively, these results suggested that H19 regulated the expression of *ITGB3* in the HTR-8 cell line, at least by partial competitive binding to let-7.

### H19 promoted HTR-8 adhesion and invasion by inhibiting miRNA let-7

**Figure 2:**
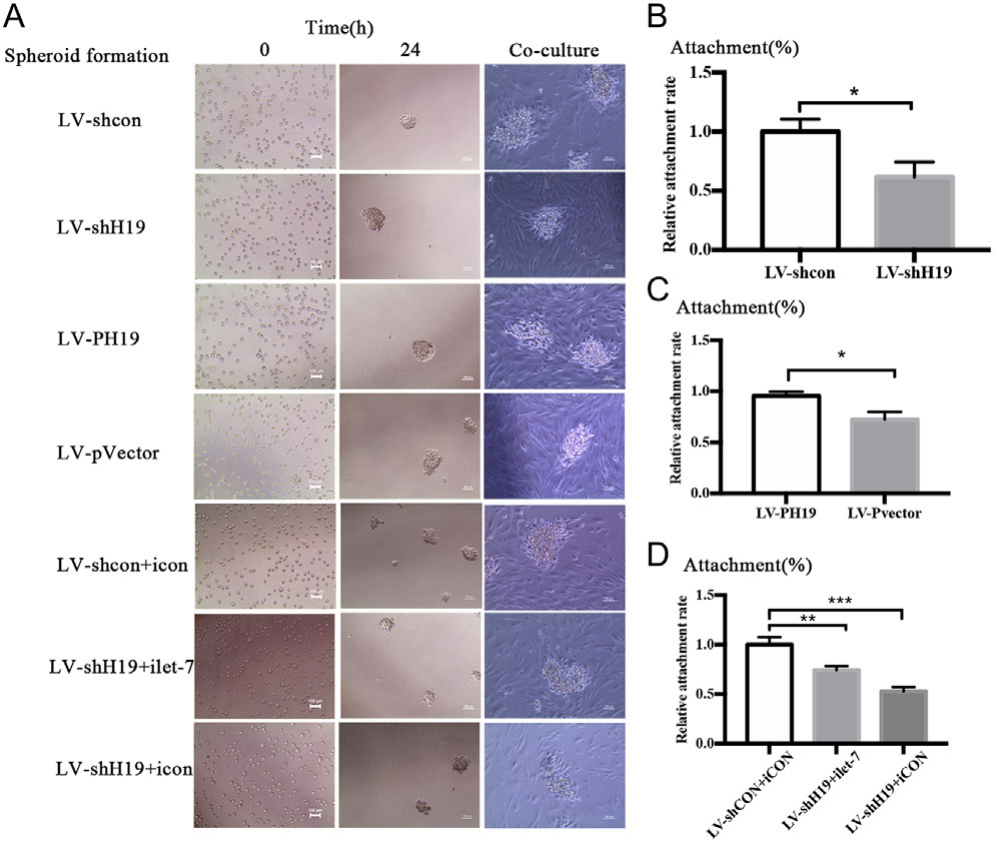
H19 promotes HTR-8 adhesion by inhibiting miRNA let-7. (A) Examples of the trophoblast spheroid and adhesion assays with HTR-8 are shown. Spheroid formation occurs under optimal conditions for the HTR-8 cells. A total of 1000 cells are seeded into an ultra-low attachment 96-well plate with a lid flat bottom in media supplemented with 5% serum and 2 mg/mL methylcellulose aggregated into compact spheroids within 24 h. Then, the trophoblast spheroids are transferred into a 6-well plate filled with HESCs, and the attachment ratio is calculated after 12 h of co-culture. The attachment ratios in the (B) H19-knockdown group, (C) H19-overexpression group, and (D) combination of H19 knockdown and let-7 inhibitor group are shown.

**Figure 3:**
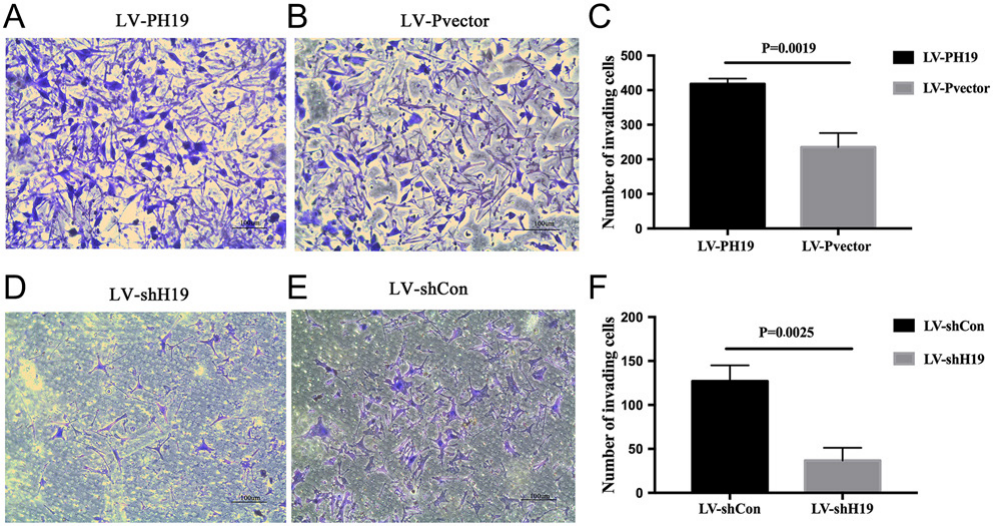
H19 promotes the invasion ability of HTR-8 by inhibiting miRNA let-7. Changes in the trophoblast invasion ability in the (A and B) H19 overexpression group and (D and E) H19 knockdown group are shown. (C and F) The statistical analysis results are shown.

The HTR-8 spheroid formation and co-culture with HESCs are described in Fig. 2A. The number of invading cells in the different lentiviral vector groups is shown in Fig. 3A, B, D and E). When H19 was downregulated by shH19, there was a decrease in cell adhesion and invasion ability (Figs 2B and 3C). To measure the change of adhesion ability, H19 was knocked down using shH19 in the presence or absence of a let-7 inhibitor; in parallel, a negative control was run. The combination of shH19 and let-7 inhibitor partially restored the adhesion ability of the HTR-8 cell to control levels (Fig. 2D). Next, H19 overexpression with LV-PH19 increased the adhesion and invasion abilities (Figs 2C and 3F). Collectively, these results suggested that H19 promoted EVT cell adhesion, and this regulation was achieved, at least in part, by reducing the bioavailability of let-7.

### miRNA let-7a directly bound to the *ITGB3* 3′-UTR region

**Figure 4:**
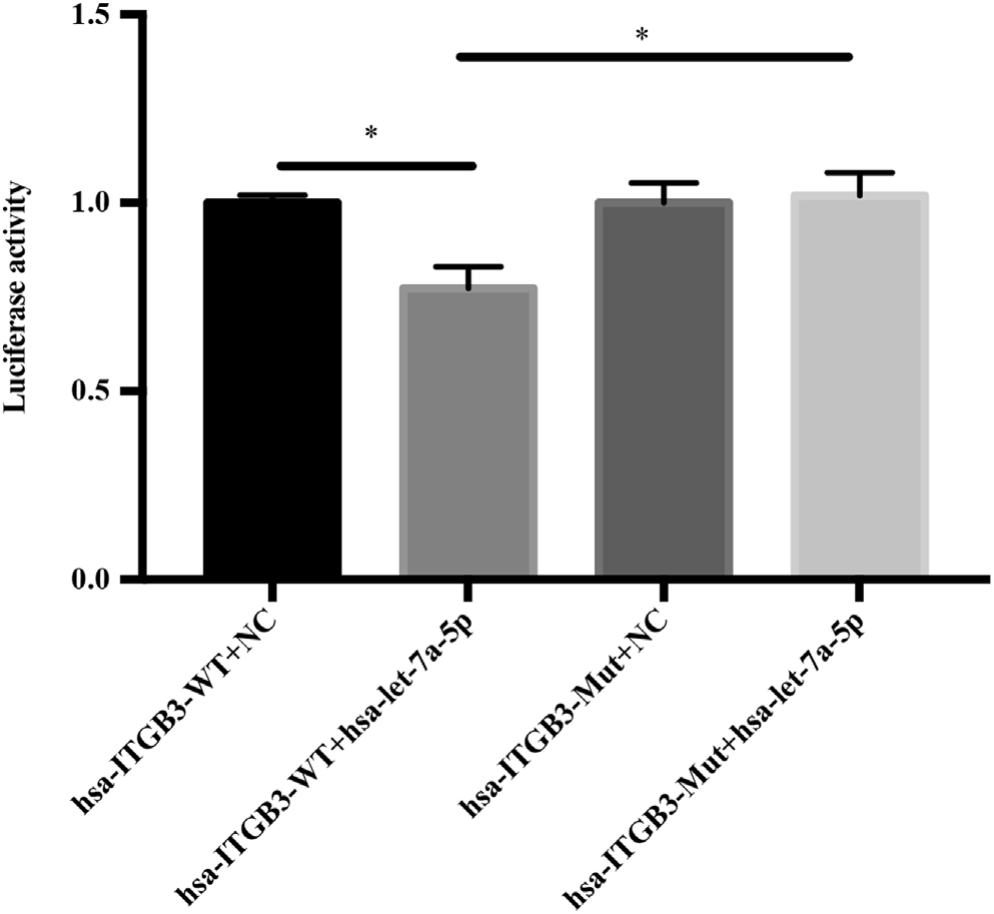
Bioinformatics prediction and luciferase reporter system detection of the target gene. Co-transfection of a wild-type integrin β3 3′-UTR reporter construct (hsa-*ITGB3*-WT) with artificial let-7a mimic hsa-let-7a-5p molecules into 293T cells results in an approximately 23% downregulation of luciferase activity, compared with co-transfection of the 3′-UTR reporter construct with the negative control. Co-transfection of human mutant integrin β3 3′-UTR reporter construct (has-*ITGB3*-MUT) with hsa-let-7a-5p results in a returned phenomenon, compared with co-transfection with hsa-*ITGB3*-WT and hsa-let-7a-5p.

miRNA let-7a-5p was hypothesized to modulate the expression of ITGB3 by binding to a specific sequence in the 3′-UTR of the *ITGB3* mRNA. A 3536-bp fragment of the 3′-UTR of the ITGB3 transcript was amplified and ligated into the pmiR-RB-REPORT vector to determine whether the expression of *ITGB3* was regulated by let-7a-5p. The hsa-*ITGB3*-WT group showed a downregulated fluorescence, compared with that of the hsa-*ITGB3*-MUT group in the 293T cell line (*P* < 0.05, Fig. 4). Compared with NC, hsa-let-7a-5p significantly downregulated the fluorescence of hsa-*ITGB3*-WT, and the reporter fluorescence in the mutant vector had a recovery effect. This result indicated that hsa-let-7a-5p was likely to interact with the 3′-UTR of this segment of the *ITGB3* gene.

### Expressions of H19 and *ITGB3* decreased in the human embryonic chorion tissue of SA

**Figure 5:**
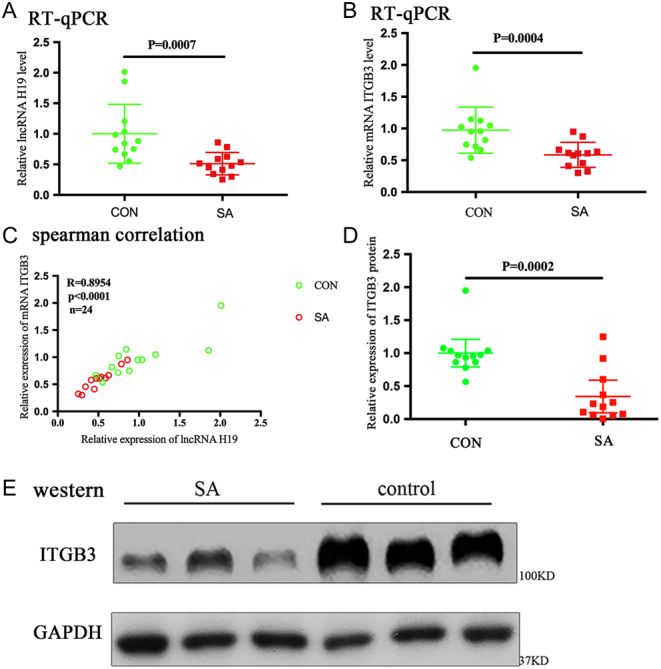
The expressions of H19 and integrin β3 (*ITGB3*) decrease in human embryonic chorion tissue of spontaneous abortion. (A and B) Scatter plot of the RNA levels on RT-qPCR shows that the relative expressions of H19 and *ITGB3* significantly decrease in the SA group. The horizontal line represents the group median, and the whiskers represent the interquartile range. (C) Spearman correlation suggests a significant *in vivo* positive correlation between the expressions of H19 and *ITGB3*. The Spearman correlation coefficient, *P* values, and sample numbers are indicated on the upper left of the plot. (D) Scatter plot of the protein levels on Western blot analysis is shown. The horizontal line represents the group median, and the whiskers represent the interquartile range. (E) The relative expression level of *ITGB3* protein in the two groups is shown. GAPDH is used as an internal control.

A total of 24 patients were enrolled in this study. Of these, 12 patients with an SA were included in the case group, whereas 12 patients who requested for elective termination of pregnancy were included in the control group. All the tissues obtained from each sample were pathologically examined. The baseline characteristics, such as age, BMI, gestational sac size, menopause day, history of pregnancy and childbirth, were equally distributed between the two groups (Table 2). The mRNA and protein expressions of H19 and *ITGB3* were reduced in the case group. Spearman correlation analysis showed a positive correlation between the expressions of H19 and *ITGB3* at the RNA level (Fig. 5).

**Table 2:** Baseline data characteristics comparison of SA group and control group.

Variables	CON (*n* = 12)	SA (*n* = 12)	*P*
Maternal age (year)	24.58 ± 3.26	24.41 ± 2.02	0.8817
Gravidity (year)	1 ± 0.58	1.25 ± 0.45	0.2629
Last menstrual period (day)	53.58 ± 5.46	52.9 ± 6.33	0.4971
Gestational sac size (mm^3^)	19.92 ± 2.54	18.08 ± 1.68	0.2088
Maternal body mass index (kg/m^2^)	17.97 ± 0.81	18.45 ± 0.43	0.2727

A *P* value of <0.05 indicated statistical significance.

## Discussion

In the present study, we showed that H19 knockdown decreased the expression of *ITGB3* by acting as a molecular sponge that enabled let-7a-5p to avoid mRNA degradation on its target gene *ITGB3*, leading to the impaired adhesion and invasion of EVT cells. In addition, the expression of *ITGB3* was partially rescued after *in vitro* transfection of the let-7 inhibitor in HTR-8 cells. Furthermore, the expressions of lncRNA H19 and *ITGB3* in embryonic chorionic tissues at 6–10 weeks of gestation were significantly downregulated in the SA group than in the control group. The expression of H19 had a significant positive correlation with ITGB3 expression, indicating that dysregulation of the H19/let-7/ITGB3 axis correlated with early SA.

Previous studies focused on the *ITGB3* function as an endometrial receptivity molecule during embryo implantation in the mid-luteal phase endometrium (Liu *et al.* 2013). *ITGB3* had been recognized as a sign of the opening of the endometrial implantation window (Haouzi *et al.* 2009). An *in vitro* experiment showed that blastocyst attachment was significantly reduced after transfection with ITGB3 siRNA (Kaneko *et al.* 2011). In addition, *ITGB3* was vital in the progression and invasion of melanoma (Muller & Bosserhoff 2008). Knockdown of calreticulin modulates the N-glycosylation of integrin β1 in HTR8/SVneo cells, thereby suppressing invasion ability in early pregnancy (Yamamoto *et al.* 2017). Some researchers suggested that the blastocyst trophoblast ectoderm undergoes partial epithelial–mesenchymal transition (EMT), including an increase in the expression of interstitial mucin, for the invasiveness of the blastocysts. A previous study showed that after 22 days of pregnancy, the expression of EMT markers, such as ZEB1, ZEB2, Twist and Snail, in the trophoblast layer increased significantly along with increase in the expressions of the ITGB3 and ITGB8 proteins (Yamakoshi *et al.* 2012). The finding indicated that *ITGB3* might act as an adhesive molecule to promote the adhesion, invasion and migration abilities of embryonic trophoblasts for the maintenance of early pregnancy.

In view of the fact that some patients experience unexplained SA in early pregnancy, we consulted the relevant literature. A significantly reduced expression of ITGB3 had been found in the endometrium of women with unexplained recurrent pregnancy loss (Germeyer *et al.* 2014); this highlighted the importance of this adhesion protein for implantation. Moreover, downregulation of ITGB3 was found in the secretory-phase endometrium of subjects with recurrent miscarriages and might have affected the early stage of embryo–endometrial interaction (Othman *et al.* 2012). PLA1/PLA2 polymorphism of the *ITGB3* gene may responsible for the increased risk of early fetal loss (Ruzzi *et al.* 2005). Previous literature reported that the receptors of integrin αVβ3 that was detected in trophoblasts may have promoted the adhesive and migratory behaviors of the trophoblasts (Kaneko *et al.* 2011, Ho *et al.* 2012, Antoniotti *et al.* 2018). The current research was conducted to focus on the effect of endometrial ITGB3 expression on abortion, while the effect of trophoblast ITGB3 expression on abortion is rare.

H19 is a multifunctional lncRNA that had been previously demonstrated to act as a molecular sponge for the major let-7 family of miRNAs, which are known to play important roles in diverse physiologic and pathologic processes (Kallen *et al.* 2013). A previous study revealed that pregnancy through assisted reproduction methods may result in the abnormal methylation of H19, which may account for the incidence of human SA (Zheng *et al.* 2013). In rat spermatozoa, the presence of insulin-like growth factor 2-H19 locus-specific DNA methylation was associated with rat embryo postimplantation loss (Pathak *et al.* 2009). In this present work, we showed that a decrease in H19 correlated with the adhesion and invasion abilities in HTR-8 cells and that the expression of ITGB3 can be partially restored by combined *in vitro* transfection of shH19 and let-7 inhibitor. The H19 being a multifunctional lncRNA may be one of the reasons for these results.

In conclusion, the *ITGB3* was verified by the dual-luciferase reporter gene to be a direct downstream target gene of the miRNA let-7 (Muller & Bosserhoff 2008). H19 may inhibit the effect of let-7 by acting as a molecular sponge to avoid mRNA degradation, thereby upregulating the expression of *ITGB3*, which can subsequently enhance EVT adhesion to the endometrium and the invasion process in the maintenance of early pregnancy.

There were several limitations that should be underscored in our study. First, the inclusion of women with BMI below 18.5 may have limited the generalizability of our results to a large population. Second, due to the drawbacks of the study design, the effect of the trophoblastic spheroid size on the efficiency of adhesion could not be ascertained.

## Declaration of interest

Nenghui Liu was involved in the project designing, project development and manuscript writing. Dongmei He and Hong Zeng contributed to the project designing, project development, data collection, data analysis and manuscript writing. The other authors have nothing to disclose.

## Funding

This study was funded by the National Natural Science Foundation of China (http://www.nsfc.gov.cn/) (grant number 81571441).

## Ethics approval

All procedures involving human participants were in accordance with the ethical standards of the institutional or national research committee. All patients provided written informed consent and ethical consent, and the project was approved by the Ethics Committee of Xiangya Hospital.
